# Entomophagy and entomo-therapeutic practices in a mountainous territory in southeast Guangxi Zhuang Autonomous Region, China

**DOI:** 10.1186/s13002-024-00700-0

**Published:** 2024-06-11

**Authors:** Huimin Luo, Chuanyin Dai, Ping Feng

**Affiliations:** 1 Key Laboratory of Ecology of Rare and Endangered Species and Environmental Protection (Guangxi Normal University), Ministry of Education, 1 Yanzhong Road, Guilin, 541006 China; 2 Guangxi Key Laboratory of Rare and Endangered Animal Ecology, Guangxi Normal University, Guilin, 541006 China

**Keywords:** Cockroaches, Edible insects, Medicinal insects, Traditional knowledge, Wasps

## Abstract

**Background:**

Although China has a long history of using insects as food and medicine and has developed numerous associated knowledge and practices, especially in its rural and mountainous areas, systematic surveys concerning this subject are limited. In-depth ethnobiological research is needed to compile a comprehensive database of edible and medicinal insects and record the associated knowledge of these food and medicinal resources.

**Methods:**

Data on edible and medicinal insects and associated knowledge about them were collected by interviewing 216 local villagers in a mountainous territory in southeast Guangxi Zhuang Autonomous Region, China.

**Results:**

Local villagers used at least 16 edible and 9 medicinal insects, of which 4 wasp species were used in both entomophagy and medicinal practices. *Parapolybia varia*, *Polistes olivaceus,* and *Anomala chamaeleon* were newly recorded edible insects in China. The wasps, *Euconocephalus* sp., *Gryllotalpa orientalis*, and *Cyrtotrachelus longimanus,* were preferred and culturally important edible insects. Populations of *Euconocephalus* sp. and *G. orientalis* were reported to have substantially decreased in recent years. Wasps and a bamboo bee were used to treat rheumatism, while cockroaches and antlions were used to treat common cold symptoms in infants. Insect-related knowledge was positively correlated with the interviewees’ age.

**Conclusions:**

Villagers have accumulated considerable local and traditional knowledge of entomophagy and entomo-therapeutic practices. However, this knowledge is in danger of being lost, which highlights the urgent need to document this information. Edible insects enrich local diets, and a more sustainable supply (such as through insect farming) could maintain local entomophagy practices. Medicinal insects are a part of local folk medicine, and pharmacological and chemical techniques could be applied to identify various biologically active substances in these insects.

## Background

Edible insects are rich in diverse nutrients, such as essential amino acids and fatty acids, and can be appetizing [1, 2]. They are used to be an integral part of the local daily diet in many cultures, especially in traditional communities and poor rural populations in underdeveloped and developing countries [3–5], although insects as a food item are becoming less popular in recent years due to westernization [6]. Insects have been regarded as an excellent alternative protein source for humans due to their high-protein content and quality [7, 8]. Furthermore, insect farming can provide the equivalent amount of animal proteins with less land and water use while emitting substantially fewer greenhouse gases [9, 10]. Unlike conventional meat production, insects have higher feed conversion efficiencies and can transform low-value organic by-products into high-quality proteins [11]. In addition, edible insects could replace fish meal and soya, which are a potential protein source for feeding livestock and aquatic animals [4, 11]. Edible insects even contain bioactive substances such as chitin and antibiotic peptides that can enhance animal immunity, reducing the use of antibiotics in conventional meat production [12]. Globally, approximately 2,111 insect species have been documented as edible [13]. Thus, edible insects (for both food and feed) can play important roles in food sustainability and security [1], as has been suggested as early as in 1975 [14].

Insects also play critical roles in traditional medicine [15, 16]. Approximately 70%–80% of the world’s rural population rely on traditional medicine for their primary healthcare, which includes various entomo-therapeutic practices [17, 18]. For example, crickets, katydids, and some grasshopper species have been recommended for illnesses of the throat and ears [18]; ants have been used to treat paralysis, gastrointestinal ailments, severe colds, pain, arthritis, gynecological disorders, mumps, asthma, insect stings and bites, dizziness, impotence, rheumatism, chickenpox, and bronchitis [16, 18]; and honey has been reportedly used to treat burns, skin disorders, respiratory, gastrointestinal, and cardiovascular illnesses and promote wound healing [19, 20]. Beetles have been used to lessen or bring down kidney pain, rheumatism, gout, ear and tooth aches, snake and dog bite sufferings, and urinary problems [16]. Vespine wasps claimed to possess anticancer properties and compounds that can stimulate the heart and kidneys [21]. As such, the medicinal interactions between humans and insects have recently aroused the interest of researchers who record these interactions and search for compounds exhibiting pharmacological activity [18, 22, 23]. To date, approximately 1,000 species with therapeutic value have been reported worldwide, although the actual number may be considerably higher due to insufficient attention paid to this field of inquiry [16].

China has a long history of using insects as food and medicine [22, 24]. Currently, at least 324 and 400 species have been documented for use as food and in traditional Chinese medicine, respectively [10, 25]. The edible insects belong to 11 orders, among which Lepidoptera, Coleoptera, Hymenoptera, and Orthoptera account for 84.56% [10]. The insects documented in Chinese pharmacopeia are distributed among 14 orders, most of which are from Blattaria, Mantodea, Homoptera, Hemiptera, Coleoptera, Lepidoptera, Diptera, and Hymenoptera [25]. However, the number of edible and medicinal insects could be underestimated in China due to the limited attention paid to this subject. Furthermore, the few relevant investigations that have been carried out were primarily conducted in regions such as Yunnan and Guizhou Provinces, which are inhabited by minority ethnic groups [12]. Entomophagy and the use of insects as medicine are a common practice across China, especially in mountainous areas (Personal Observations). Hence, there may be a large number of edible and medicinal insect species awaiting documentation in these regions. Moreover, the investigational approach adopted by previous researchers may have low estimates of edible and medicinal insects due to, for example, the use of anecdotal records and a lack of systematic surveys, and also the inadequate linguistic fluency of especially foreign investigators. In-depth ethnobiological research is thus needed to compile a comprehensive database of edible insects, including medicinal insects, and record the associated knowledge of these foods and medicinal resources in China.

**Fig. 1:**
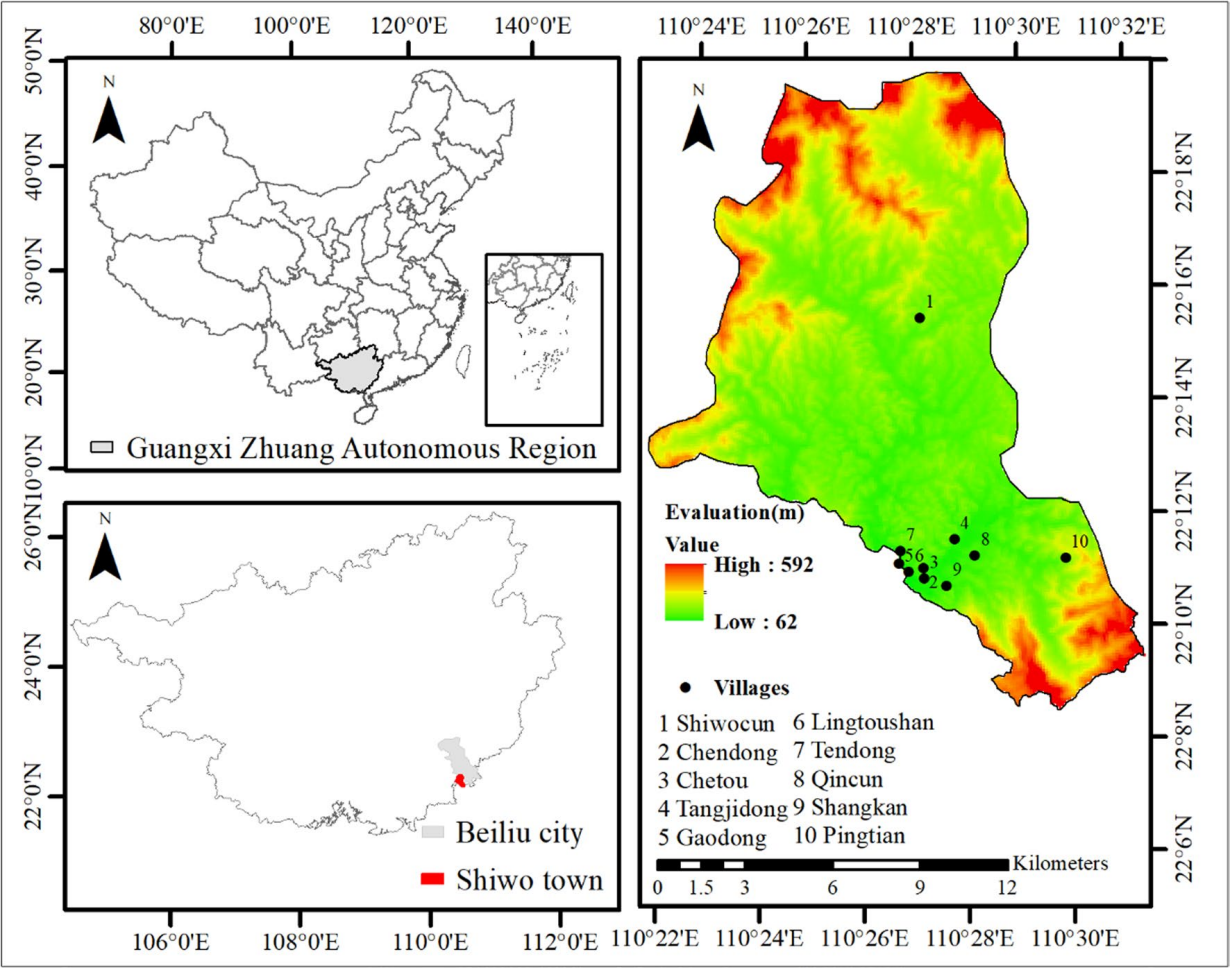
Map showing the location of the study area

In this study, we carried out an ethno-entomological study in a mountainous area in the district of Beiliu City. The city is located in the southeast of Guangxi, Zhuang Autonomous Region **(**ZAR), China (Fig. 1), a well-known tourist location characterized by natural sights and local cultures. The percentage of forest coverage is up to 66.35%, while the vegetation consists of natural evergreen broad-leaf forests and numerous plantations [26]. The complex and varied geographical environment and the subtropical monsoon climate have created suitable conditions for generating and maintaining rich biodiversity. It has 989 known species of wild vascular plants, including abundant nectariferous and medicinal ones [26, 27]. Although there are few scientific reports on local animal resources, 294 vertebrates have been documented in one of its protected areas, the Darongshan Natural Reserve [28]. There are 22 species of national, second-class, key protected animals and 62 species of Guangxi ZAR key protected animals, most of them birds, reptiles, amphibians, and mammals [29, 30].

The rural population constitutes 50.52% of the total population of approximately 1.22 million in Beiliu City. In rural areas, people primarily obtain basic living materials and income by planting crops and raising poultry. Due to long-term contact with the natural environment, local communities have developed traditional practices that incorporate local floral and faunal resources to survive, such as collecting wild honey, beekeeping, using plant-based medicine, eating wild edible plants, and using vertebrates and invertebrates as food and medicine. It is necessary to record and understand traditional knowledge concerning the use of local biological resources from an ethnobiological perspective, as it has paramount significance for both the protection of traditional culture and the sustainable use of local biological resources [31, 32].

As such, the goals of this study were to: (1) document the diversity and relevant knowledge about insects used in the local daily diet and folk medicine; (2) identify the factors influencing insect-related knowledge or practices; and (3) provide a few insights for promoting the use of edible and medicinal insects.

## Methods

### Study area

A field survey was conducted in the district of Shiwo town south of Beiliu City (Fig. 1). It is a mountainous region with a territory of approximately 158.26 km^2^. The altitude ranges from 50 to 830 m, and the percentage of the forest coverage is up to 64%. The average annual precipitation is 1,534.9 mm, and the average annual temperature is 24 °C [33]. There are approximately eighty thousand inhabitants. All are ethnic Han Chinese. The locals are primarily engaged in agricultural production, or are migrant workers. Rice, tuberous and legume crops, corn, and sugarcane are the main planting crops. Spice plants, fruit forests, ornamental trees, and medicinal herbs constitute the main economic planting.

### Field survey and data collection

We conducted a field investigation in the study area from July to August 2022. Semi-structured interviews, key informant interviews, and participatory investigations were carried out [34]. We interviewed 216 locals in 10 villages (Table 1), including 112 males and 104 females aged 16 to 88. The villages were selected using convenient sampling. Local villagers speak local Chinese (Shanglihua), without any religious belief reported. Most of the interviewees were selected randomly, while eight informants who were experienced in making medicinal liquor were recommended by the villagers.

**Table 1:** Geographical and sociodemographic information of the interviewed villages

Village	Longitude(E)/Latitude (N)	Altitude (m)	Age	Male/Female	Occupation	Ecological feature
Shiwocun	110.463/22.261	116	18–82	23/19	Farmer, businessman, mechanic, migrant worker, factory worker	All the villages host flat terrain conducive to agricultural practice. The soil are mainly paddy soil and latosol red soil. Rice, tuberous crops, legume crops, corn and sugarcane are main crops. *Litchi chinensis* and *Phellodendron chinense* are widely planted. Pingtian is an important planting area for *Illicium verum*
Chengdong	110.459/22.188	84	16–83	8/8	Farmer, migrant worker, factory worker, shopkeeper	
Chetou	110.459/22.185	82	26–62	6/5	Farmer, factory worker, migrant worker, shopkeeper	
Tangjidong	110.469/22.195	74	23–78	13/5	Farmer, teacher, migrant worker, factory worker	
Gaodong	110.451/22.189	66	26–85	11/9	Farmer, teacher, migrant worker, factory worker	
Lingtoushan	110.454/22.187	71	23–87	5/9	Farmer, factory worker, migrant worker	
Tendong	110.452/22.193	66	16–84	11/8	Farmer, businessman, mechanic, migrant worker, Factory worker	
Qincun	110.476/22.190	78	17–78	13/11	Farmer, factory worker, migrant worker	
Shangkan	110.466/22.182	72	17–88	8/12	Farmer, businessman, factory worker, mechanic, migrant worker	
Pingtian	110.504/22.187	184	21–75	14/18	Farmer, factory worker, migrant worker	

At the beginning of the interviews, each respondent was informed about the objective of the survey and encouraged to participate on a volunteer basis with verbal consent. Each interview was conducted face-to-face in local Chinese at their homes or in the field. The sociodemographic information of the interviewees was recorded, including gender, age, educational background, and livelihood (Table 2). The respondents were asked whether they had consumed insects, and if yes, they were further asked to list the species and include related morphological, behavioral, and residential characteristics. Participants were further asked to describe the motivation behind their consumption, the collection practices and preparation methods for each species mentioned, and to provide their opinion on the population size of each edible insect. Non-consumers were asked whether they knew about edible insects, and whether they accepted insects as food, and the reasoning behind their choice. All of the respondents were asked whether they knew about medicinal insects. Relevant information—including insect collection methods, parts used, diseases treated, and detailed therapeutic practices—was documented.

**Table 2:** Characteristics of the respondents

Demographic features	Categories	N^1^ (216)	N^2^ (207)	N^3^ (205)	N^4^ (137)	Number of species mentioned (Mean ± SD)
Age	16–29	44	40	40	11	7.25 ± 3.00
	30–39	14	13	12	5	6.92 ± 1.80
	40–49	21	21	20	15	10.57 ± 2.77
	50–59	53	52	52	37	12.19 ± 3.09
	60–69	44	41	41	32	11.98 ± 3.52
	≥ 70	40	40	40	37	13.83 ± 2.64
Gender	Male	112	110	109	62	10.75 ± 3.82
	Female	104	97	96	75	11.31 ± 3.83
Education	No education	17	15	15	12	13.33 ± 2.77
	Primary school	123	121	121	96	12.26 ± 3.17
	High school	63	60	58	22	8.22 ± 3.71
	Junior college or above	13	11	11	7	9.36 ± 3.47
Livelihood	Formal employment	51	49	49	20	8.57 ± 3.07
	Informal employment	17	15	15	14	13.6 ± 3.33
	Farmer	76	73	72	61	12.3 ± 3.13
	Other	72	70	69	42	10.83 ± 4.15

N^1^: number of interviewees. N^2^: number of respondents mentioned the edible or medicinal insects; N^3^: number of respondents consuming the edible insects; N^4^: number of respondents mentioned the medicinal insects

Each interview lasted between 20 min and one hour. All the information was recorded with a pen on the spot. With the assistance of local villagers, field walks were conducted to search for the insects mentioned in the interviews. The walks were also used to observe the insects’ behavioral characteristics, and to collect specimens and photographs to verify the taxonomic identities of the species. The species were identified with the help of the book “*Wasps Fauna of Yunnan*” [35], Global Biodiversity Information Facility (GBIF) (https://www.gbif.org/), and Flora Reipublicae Popularis Sinicae (http://www.iplant.cn/frps). As a few frequently mentioned insect species were not found in the field surveys, we looked for and downloaded photographs from the Global Biodiversity Information Facility (GBIF) (https://www.gbif.org/) according to the species characteristics provided by the respondents in order to identify their identities with the respondents as possible.

### Data analysis

The verbal interview data were documented and categorized, and we explored the salient aspects of the interviewees’ speech. The nonparametric Spearmen correlation was used to examine the relationship between the number of listed insects and the respondent’s age and education. To detect the effect of gender and livelihood on the number of species listed by interviewees, the Mann–Whitney U test and Kruskal–Wallis test were conducted, respectively.

Based on the oral recounting of lists of edible species collected in the interviews, Smith’s salience index (S) was used to determine the cultural importance of edible species in the study area: S = ∑((L-R_j_ + 1)/L)/N, where L = the number of items in a list; R_j_ = the rank of the item in the list (first = 1); and N = the number of lists in the sample [36, 37]. This index considers both the frequency and rank of items in the list (prominence, familiarity, and representativeness) [32]. Items mentioned earlier and more frequently have a relatively higher mean percentile rank, indicating their higher cultural importance. For example, an item mentioned first in every list would have an index of 100, whereas items occurring later in lists or not at all in some lists would have indexes that decline toward zero [38].

The informant consensus factor (ICF) was used to determine the relative importance of medicinal insects for the treatment of different diseases: ICF = (Nur − Nt)/(Nur − 1), where Nur is the number of mentions for each disease, and Nt is the total number of medicinal insect species used for a given disease by all informants [39]. ICF values range from 0 to 1, where a value close to 1 indicates high consensus among informants on using an insect to treat a specific disease [39–41]. ICF can highlight widely used insects that merit further pharmacological and phytochemical investigation [40].

## Results

### Species diversity

**Fig. 2:**
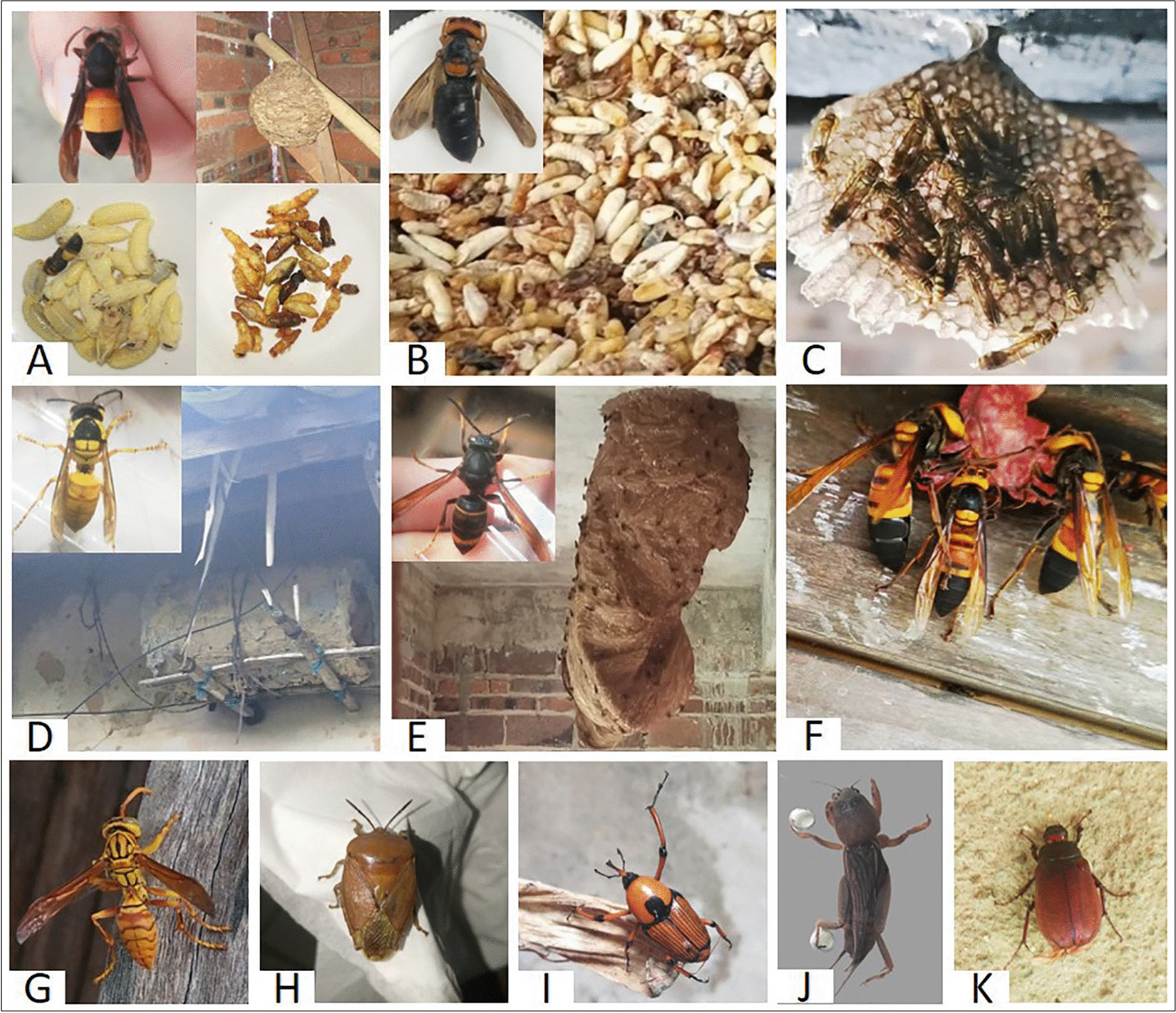
Edible insects collected in the study area (Photos are not scaled). **A**. The nest, larvae, pupae, and adult of *Vespa affinis*. **B**. *Vespa basalis*. **C**. *Parapolybia varia* in house. **D**. *Vespa bicolor*. The wasp invaded and built a nest in an artificially cylindrical beehive. **E**. *Vespa velutina* and its nest hanging on the ceiling. **F**. *Vespa ducalis*. **G**. *Polistes olivaceus*. **H**. *Tessaratoma papillosa*. **I**. *Cyrtotrachelus longimanus*. **J**. *Gryllotalpa orientalis*. **K**. *Anomala chamaeleon*

**Fig. 3:**
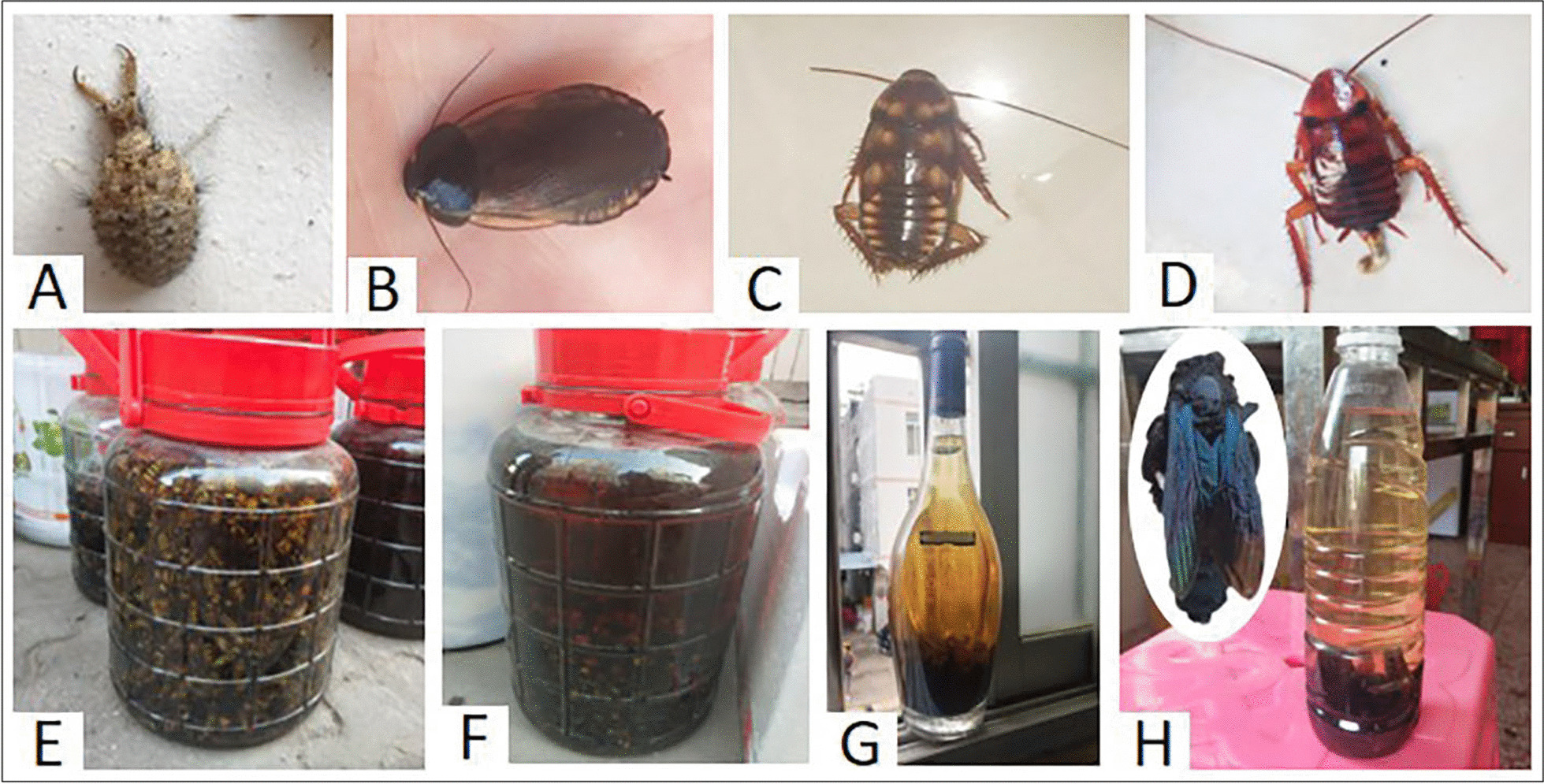
Medicinal insects and medicinal liquor collected in the study area (Photos are not scaled). **A**. Antlion. **B**. *Pycnoscelus surinamensis*. **C**. The larva of *Periplaneta australasiae*. **D**. The larva of *Periplaneta americana*. **E**. The medicinal liquor of *Vespa bicolor*. **F.** The medicinal liquor of *Vespa ducalis*. **G**. The medicinal liquor of *Vespa affinis*. **H**. The medicinal liquor of *Xylocopa dissimilis*

We recorded 16 consumed insects belonging to 5 orders and 10 families (Table 3 and Fig. 2). Nine medicinal insect species were also documented, belonging to three orders and five families (Table 4 and Fig. 3). Four wasp species were used both in entomophagy and medicinal practices, where the adult was used for medicine, and the larvae and pupae used for food. However, of the 21 species documented, only 16 species have specimens and 15 species were successfully identified (except an antlion, a larva of Myrmeleontidae). These specimens were deposited in Guangxi Normal University. *Samia ricini* and *Bombyx mori* were identified without voucher specimen as they were important and widely raised cash insects in China in the past. *Euconocephalus sp.* was confirmed through photographs. Two unknown species were locally called Niujiaolang (a wasp) and Mujichong (a scarab larva), both of which were not found in the field and confirmed via photographs.

**Table 3:** Edible insects consumed in the study area

Order	Family	Species	Vernacular name	Harvest season	Part consumed	Cuisine	Salience Index (S)	Mentions	Consumers
Hymenoptera	Vespidae	*Vespa bicolor* Fabricius, 1787	Huangjiangfeng	Summer, autumn	Larvae, pupae	Frying	0.36	166	165
		*Vespa ducalis* Smith, 1852	Baiyizhuo	Summer, autumn	Larvae, pupae	Frying	0.18	36	36
		*Vespa affinis* Linnaeus, 1764^a^	Guanfeng/Tanfeng	Summer, autumn	Larvae, pupae	Frying	0.35	192	191
		*Vespa basalis* Smith, 1852^a^	Guanfeng/Tanfeng						
		*Vespa velutina* Lepeletier, 1836^a^	Huangjiao/Guanfeng/Tanfeng						
		Unknown (A wasp with whole black body)	Niujiaolang	Summer	Larvae, pupae	Frying	0.26	138	137
	Polybiidae	*Parapolybia varia* Fabricius, 1787	Huayaofeng/Niuerfeng	Summer	Larvae, pupae	Frying	0.42	190	189
	Polistidae	*Polistes olivaceus* Deg., 1773	Changjiaotuo	Spring, summer	Larvae, pupae	Frying	0.34	192	191
Hemiptera	Tessaratomidae	*Tessaratoma papillosa* Drury, 1770	Jiuweixie	Summer	Nymphs (fifth instar), adults	Frying, baking	0.6	90	90
Lepidoptera	Saturniidae	*Samia ricini* Jones, 1791	Mushucan	Spring, summer	Pupae	Frying	0.63	98	98
	Bombycidae	*Bombyx mori* Linnaeus, 1758	Canchong	Spring, summer	Pupae	Frying	0.63	98	98
Orthoptera	Gryllotalpidae	*Gryllotalpa orientalis* Burmeister, 1838	Pagou	Spring	Adults	Frying	0.85	191	190
	Tettigoniidae	*Euconocephalus* sp.	Hexia	Summer	Adults	Frying	0.96	193	192
Coleoptera	Curculionidae	*Cyrtotrachelus longimanus* Fabricius, 1775	Sanmihuang	Spring, summer	Larvae, adults	Frying	0.75	143	143
	Scarabaeidae	*Anomala chamaeleon* Fairmaire, 1887	Huangchong	Spring	Adults	Frying	0.76	129	129
		Unknown (A larva of scarab. The larvae were collected from the planting areas of peanuts, cassava and sweet potatoes.)	Mujichong	Spring	Larvae	Frying	0.58	29	29

^a^The three species share same values since they have a same local name

**Table 4:** Medicinal insects used in the study area

Order	Family	Species	Vernacular name	Part used	Mentions	Therapeutic practices	Nur	Nt	ICF
Neuroptera	Myrmeleontidae	Unidentified (Antlions, the larva of Myrmeleontidae)	Shawochong	Larvae	120	Antlions and cockroaches are used alone or in combination. These live insects were disinfected in boiling water, then pounded, and mixed with a little warm water and were drunk to treat common cold symptoms in infants	137	4	0.98
Blattaria	Blaberidae	*Pycnoscelus surinamensis* Linnaeus, 1758	Dizhang	Larvae, adults	83				
	Blattidae	*Periplaneta americana* Linnaeus, 1758	Jiaozhang	Larvae, adults	135				
		*Periplaneta australasiae* Fabricius, 1775	Jiaozhang	Larvae, adults	135				
Hymenoptera	Apidae	*Xylocopa dissimilis *Smith, 1857	Zhutongfeng	Adults	4	Live adults were used to make medicinal liquors to treat rheumatism	8	5	0.43
	Vespidae	*Vespa bicolor* Fabricius, 1787	Huangjiangfeng	Adults	8				
		*Vespa ducalis *Smith, 1852	Baiyizhuo	Adults	8				
		*Vespa affinis* Linnaeus, 1764	Guanfeng/Tanfeng	Adults	8				
		*Vespa basalis* Smith, 1852	Guanfeng/Tanfeng	Adults	1

Almost each of the species has a local name (Table 3 and Table 4). However, the criterion adopted by the local villagers was unclear for distinguishing the wasps, as the name ‘Guanfeng’ or ‘Tanfeng’ was assigned to *Vespa affinis*, *Vespa basalis,* and *Vespa velutina,* possibly due to locals perceiving similar morphological and behavioral characteristics, such as crock-shaped nests and black bodies. Only a few respondents (n = 3) could distinguish *V. velutina* from Guanfeng and called it ‘Huangjiao’ as the species has yellow feet.

### Respondents’ characteristics and insect use

In total, 207 interviewees (95.8%) had mentioned edible or medicinal insects, of which 205 had consumed insects and 137 mentioned the medicinal species (Table 2). A positive relationship was observed between the number of listed insects and the respondent’s age (*r* = 0.606, *n* = 207, *p* < 0.001), while a negative correlation was found between the number of listed insects and educational achievement (*r* = -0.476, *n* = 207, *p* < 0.001). There was also a significant association between the number of listed insects and livelihood (*x*^*2*^ = 38.776, *df* = 3, *p* < 0.001). Individuals with a stable income (formal employment) mentioned fewer insects. However, no significant association was found between gender and the number of listed insects (*p* = 0.11).

A few respondents (n = 11) had never consumed insects and had negative attitudes toward entomophagy, except a teenager who was willing to try them. Some of these respondents (n = 4) stated that “Insects have no value, but during times of hardship, people would be too hungry and would eat them.” Others (n = 6) said that the negative psychological effects caused by the appearance or behavior of insects, such as fear and nausea, made them unacceptable as food. However, among these respondents, one man (48 years old) was able to list Guanfeng, and a woman (34 years old) was able to list *Euconocephalus* sp., *Gryllotalpa orientalis*, *Parapolybia varia*, Guanfeng, *Vespa bicolor*, *Polistes olivaceus*, and Niujiaolang, because they had collected these insects for family consumption.

### Cultural importance of edible insects

The palatability of insects was the main reason mentioned for their consumption. Consumers did not perceive entomophagy as a means of dealing with starvation or for nutritional supplementation. They regarded edible insects as delicacies, consuming whole insects as snacks and dishes. Among the documented species (Table 3), *Euconocephalus* sp. was consumed most frequently, followed by *P. olivaceus*, *G. orientalis*, *P. varia*, Guanfeng (*V. affinis*, *V. velutina*, *V. basalis*), and *V. bicolor*.

The most culturally salient insect was *Euconocephalus* sp. (S = 0.96), followed by *G. orientalis* (S = 0.85), *Anomala chamaeleon* (S = 0.76), *Cyrtotrachelus longimanus* (S = 0.75), silkworms (*Samia ricini* and *Bombyx mori*) (S = 0.63), *Tessaratoma papillosa* (S = 0.6), and Mujichong (S = 0.58). The values were lower than 0.5 for the other species (Table 3). The order Coleoptera was the most culturally important, reaching the highest total salience index (S = 2.09), followed by Hymenoptera (S = 1.91), Orthoptera (S = 1.81), Lepidoptera (S = 0.63), and Hemiptera (S = 0.6).

### Edible insect collection and preparation

All of the edible insects recorded in this survey were harvested in the wild except *S. ricini* and *B. mori*, which locals reared. We recorded the collection and preparation practices according to taxonomic groups.

#### Wasps

Local inhabitants were familiar with suitable habitats for wasps’ nests due to long-term collecting practices and life experiences (e.g., wasp stings have led locals to consciously pay attention to the locations of nests). In most cases, locals collected wasps when they hunted, grazed cattle, gathered firewood, picked wild fruits, or searched for plant medicines in the mountains. They searched for wasp nests by observing wasps’ flight direction and hearing their sounds. At other times, they would accidentally get stung first and then notice the nest nearby. However, they believed that wasps would not attack humans unless the wasps were disturbed. During summer vacations, children became important discoverers of wasps’ nests.

The locals use fire to produce smoke and high temperatures to drive out or destroy swarms. They never consumed adult wasps and regarded them as dangerous and poisonous; therefore, they only collected larvae and pupae. *P. olivaceus* (Fig. 2G) and *P. varia* (Fig. 2C) were collected from thickets, thorny bushes, and even houses, as they may build nests on ceilings, crossbeams, widows, cupboards, and other hidden areas. *P. varia* has a thin waist, and its nest resembles the ear of cattle. Their vernacular names (Huayaofeng and Niuerfeng) were used based on these noticeable characteristics. They are not an aggressive species, and minimal smoke is needed to expel the swarms from their nests. However, when stung, acute pain, partial redness, and swelling would appear and take approximately 30 min to resolve (30 mentions).

*V. affinis* (Fig. 2A), *V. velutina* (Fig. 2E)*,* and *V. basalis* (Fig. 2B) typically build nests in thorny bushes and tall trees around houses or in the forests. They also build nests on the ceilings and crossbeams of houses. The nests of *V. bicolor* (Fig. 2D) are generally found in the hollows of trees and burrows and, occasionally, in houses. Moreover, *V. affinis* and *V. bicolor* may occupy the same location year after year. Two men stated that they harvested the nests of *V. affinis* at the same location for two consecutive years, while one woman stated that she found *V. bicolor* nests on the same branch for two consecutive years. In contrast, *V. ducalis* (Fig. 2F) only builds nests underground. These five species are extremely aggressive, with venom containing toxic and allergic components [42, 43]. According to respondents (n = 62, 28.97%), after being stung, symptoms including sharp pain, numbness, skin redness, and swelling (as well as chills and dizziness if the victim was stung in the head) would appear quickly, and the symptom duration was long (at least several hours). Essential balm (a Chinese herbal oil for pain relief) is used to alleviate pain but has little effect. There are no efficient traditional therapies for stings, and sufferers rely primarily on immune ability. If a sting causes a severe allergic reaction, locals go to the hospital for treatment immediately. Although these wasps can be dangerous, the locals would not give up these delicacies. The harvesting season was from summer to autumn. The local saying “Guan (*V. velutina*, *V. basalis* and *V. affinis*) in July, Diwa (unknown) in August, Huangjiang (*V. bicolor*) in September, Baiyizhuo (*V. ducalis*) in October” lists the optimal harvesting stages for each species according to the lunar calendar. The unidentified ‘Diwa’ is a species that builds nests underground (whose name means ‘dig the ground for nests’). However, the Diwa consumers (n = 8, all over 60 years old) indicated that the species had not been observed for many years and may have disappeared from the region.

Males were the predominant wasp collectors. Some respondents (n = 6, 2.8%) stated: “It is easy to explain but hard to perform, and the biggest obstacle is fear.” The wasps were generally collected during the daytime. Collectors wore thick raincoats and rain boots or other clothes covering the entire body. Long bamboo poles, hemp rope, iron hooks, firewood, and cloth or mesh bags were used for collection. Sometimes, thick gloves, shovels, a firewood chopper, and a ladder were also needed. When a nest was built on a high branch, firewood was tied to the end of a bamboo pole and then ignited to expel the swarms. A hook connected with hemp rope was used to pull off the nest. Some respondents (n = 4, 1.86%) conducted collections at night, especially for large nests and underground nests with massive swarms. Before collection, they located the entrances of the nests and observed swarm activities in the daytime to determine the best burning position. When swarms congregated in and around the nest at night, collectors would light a fire, and the swarms would fly into it due to phototaxis. Another method mentioned by the respondents (n = 2, 0.93%) was using spider threads to block the exits of a nest at night. Then, while wearing thick gloves, collectors would take the nest using a flour bag. This method was only applied to the nests of *V. affinis*, as the entire swarm returned to the nest at night.

The larvae and pupae of these species were fried with salt, which gave them a crispy and salty taste. The species size varied, with the largest being *V. ducalis*, followed by *V. affinis*, *V. velutina*, *V. basalis*, *P. olivaceus,* and *P. varia*. Some consumers preferred larger sizes, but most consumers exhibited no size preference.

#### Hemiptera

*T. papillosa* (Fig. 2H) is a stinkbug that can eject a yellow, pungent liquid. This species is mainly collected in the summer from fruit trees, such as *Dimocarpus longan*, *Litchi chinensis,* and *Phellodendron chinense*. Harvesters collect them by hand without any protective measures. Hence, their fingers can stain yellow and need some time to recover. Some respondents (n = 6, 2.8%) believed the pungent liquid could burn their skin. The stinkbugs are placed in water to eliminate the odorous chemicals, then boiled, and fried. Alternatively, they are baked after stuffing the leaves of *Mentha cannadensis*, *Perilla frutescens*, *Ocimum basilicum,* or *Piper sarmentosum* into their abdomens. However, most interviewees disliked *T. papillosa* due to the scent chemicals and found them unacceptable as food. In addition, the stinkbugs were the main pest of fruit trees. Therefore, locals usually use pesticides to kill them rather than eat them. Many consumers of stinkbugs responded that they had stopped eating them for many years. As such, the consumption of *T. papillosa* has substantially declined.

#### Lepidoptera

The locals used to rear *S. ricini* and *B. mori* in spring and summer using the leaves of planted mulberry (*Morus alba*) and cassava (*Manihot esculenta*). They would extract the pupae and sell the cocoons. Eating fried pupae was customary until buyers indicated they wanted intact cocoons. Hence, locals gradually gave up eating the pupae as the main purpose of rearing the insects was to earn income. Currently, only a few locals have engaged in silkworm breeding, as they consider the work difficult with a low economic return. The last silkworm breeding was said to have occurred five years previously. As a result, while some consumers said they would buy the pupae to eat, none were available in local markets.

#### Orthoptera

*G. orientalis* (Fig. 2J) and *Euconocephalus* sp. were collected from paddy fields. Local inhabitants collected *G. orientalis* when they plowed and raked fields, as it lives underground, especially in fertile farmlands. They were caught by hand, with bottles or buckets used as containers. The collected insects were then rinsed with clean water, salted, and fried. *Euconocephalus* sp. was collected during summer rice harvesting. Locals also caught them by hand, pulling their wings and mandibles out to prevent them from escaping and biting. They would then be placed in a cloth bag. These insects were fried after cleaning.

#### Coleoptera

*C. longimanus* (Fig. 2I) is a primary pest of bamboo. They nibble on and tunnel into bamboo shoots, making them yellow, shriveled, and full of small holes. These characteristics were used as clues by locals to find juvenile insects. For adult *C. longimanus,* local people took advantage of their characteristic death mimicry behavior by shaking bamboo so they would fall to the ground. Another method was using sticks to knock adults out of the bamboo. Both larvae and adults of *C. longimanus* were consumed after frying or baking.

The consumption of *A. chamaeleon* (Fig. 2K) occurred in the past. Consumers of this species were all 44 years or older and explained that the species’ suitable habitat had disappeared as the local vegetation was transformed into bamboo forest approximately 35 years ago. They reported that these insects would hide in the sand in the daytime and gather in groups on the sand’s surface at night. Thus, they collected *A. chamaeleon* on the sand of the river bank at night. The insects were found using moonlight or flashlight, and bottles were used as containers. The wings of the collected insects were removed, and the bodies were then cleaned, salted, and fried. In the study area, *A. chamaeleon* was called “yellow insect,” and *Anomala cupripes* (Hope, 1839) was called “green insect.” These two species are similar in morphology except for their coloration. Local belief states that the yellow insects became green after the Qingming (sweeping tomb) Day, as *A. chamaeleon* was rare or disappeared, but *A. cupripes* emerged after this day. Locals never consumed *A. cupripes* as they claimed, “No one would eat the green insect (*A. cupripes*).”

#### Edible insect population trends

According to the respondents, *Euconocephalus* sp.*, G. orientalis,* and *A. chamaeleon* populations have declined in the study area. Most interviewees (n = 73) believed that the extensive use of pesticides has led to declines in *Euconocephalus* sp. and *G. orientalis* populations. Some (n = 3) indicated that the use of chemical fertilizer may have had a negative effect on *G. orientalis*. One farmer believed that replacing cattle with machinery for plowing farmland in winter may have adversely affected *G. orientalis* populations. As for *A. chamaeleon*, it is quite rare due to the loss of suitable habitat (129 mentions).

In contrast, wasp populations were reported to be increasing. The reasons given for this were varied. First, wasp nests were frequently observed in or around houses, especially *P. olivaceus* (15 mentions), *V. affinis* and *V. velutina* (15 mentions), and *V. bicolor* (8 mentions). Second, in the past, many locals collected wasps’ nests during the harvest seasons because they grazed their cattle and gathered firewood in the mountains nearly every day. However, in recent years, the number of cattle herders has substantially decreased as machinery is used to plow the land, while most households now use electricity and gas for cooking. In addition, many locals do not need to work in the wild as frequently as in the past. Therefore, the declining frequency of harvesting may have promoted the expansion of wasp populations (7 mentions). Third, five beekeepers stated that the frequency of wasps preying on their breeding bees and encroaching into beehives has increased in recent years.

### Entomo-therapeutic practices

#### Cockroaches and antlions

Antlions and cockroaches (*Pycnoscelus surinamensis*, *Periplaneta australasiae,* and *P. americana*) were used alone or in combination to treat common cold symptoms in infants (e.g., by concocting an elixir using approximately 10 antlions, 1–2 cockroaches, or 5 antlions mixed with 1–2 cockroaches) (Fig. 3 and Table 4). These insects were readily available in rural areas. Locals generally captured them from woodsheds and in the corners of houses. The live insects were disinfected in boiling water, pounded, mixed with warm water, and drunk.

#### Medicinal liquor

*V. bicolor*, *V. ducalis*, *V. affinis*, *V. basalis,* and *Xylocopa dissimilis* were used to make medicinal liquors to treat rheumatism (Fig. 3 and Table 4). When collected for medicinal purposes, locals wore protective clothing to catch adult wasps instead of smoking them out of nests. Because *V. bicolor*, *V. affinis,* and *V. ducalis* prey on bees, collectors also waited at apiaries and used insect nets to catch them. *X. dissimilis* lives in dry bamboo, and even bamboo poles used to hang clothes were suitable habitats for *X. dissimilis*, which could be found by the holes they made. Collectors stated that the number of *X. dissimilis* typically found in one pole was approximately 10 individuals. To collect them, they blocked the holes with net bags and drove them into the bags by hitting the bamboo or using smoke. *X. dissimilis* is an aggressive species. One woman who was stung in the arm experienced partial edema and intense pain for two days. The collectors usually directly shook these insects from containers into alcohol, although other methods include freezing the insects in refrigerators or scalding them with boiling water to render them immobile and then immediately placing them into alcohol. After a month of soaking, the infused alcohol can be drunk. However, the frequency and amount of liquor consumed must be monitored due to its side effects. Excessive drinking can cause "shanghuo," which causes patients to experience toothaches and a sore throat. Locals suggested drinking a small cup of medicinal liquor (around 15 ml) a day to treat rheumatic pain.

#### Informant consensus agreement on entomo-therapeutic practices

There was a high consensus (ICF = 0.98) (Table 4) among informants concerning the use of antlions and cockroaches to treat the common cold in infants. However, a relatively lower consensus (ICF = 0.43) was observed for treating rheumatism with medicinal liquor.

## Discussion

### Factors influencing local insect-use knowledge

In this study, the number of listed insects was positively correlated with the informants’ ages. Older adults possessed more knowledge about the use of insects than younger people. This could be attributed to the long-term experience of older adults in using insects as food and medicine. They were also more familiar with the local environment and primarily responsible for collecting insects for their families. However, no significant difference between genders was detected concerning the number of insects mentioned and used, suggesting that men and women were equally knowledgeable about the use of insects. Similar patterns of insect-related knowledge distribution have been reported in Botswana [44].

The overall educational level was low in this population, although younger people generally received more education than older adults. However, we found that insect-using knowledge was negatively correlated with education background. This may indicate that modern educational systems are not advocating for the protection and use of local traditional knowledge. Livelihood was also found to significantly affect the number of insects listed by respondents. It is likely that individuals with a stable income were less closely connected with the local natural ecosystem and, therefore, had less need for and contact with insects.

### Edible insects

At present, 324 edible insects have been systematically cataloged in China [10, 12], of which only a few edible insect species have been documented in Guangxi ZAR, including termites, mole crickets, and wasps. Although a few ethno-entomological studies have been carried out in recent years [45, 46], no new edible insect species has been reported. In this study, we documented 16 edible insects, of which two wasp species (*P. varia* and *P. olivaceus*) and a scarab (*A. chamaeleon*) were newly recorded in China. The salience index indicated that Coleoptera, Hymenoptera, and Orthoptera were culturally important orders consumed by locals. They are also the most commonly consumed orders in China and globally [1, 10]. Our results also found that the local population preferred to consume meadow grasshoppers, mole crickets, wasps, and weevils, which had high salience indices. These results are in accordance with our observation that consuming insects is a common practice in rural areas in China, and future efforts are needed to catalog these resources throughout the country, especially in mountainous ranges.

We noted that the nutritional composition of 11 species recorded in this study has been previously documented [12, 47–50], except *A. chamaeleon, P. olivaceus,* and *P. varia*. Overall, these insects contain a variety of nutrients beneficial to humans. For example, the larva of wasps (*V. velutina*, *V. affinis*, *V. bicolor*, and *V. basalis*), silkworm pupae (*B. mori* and *S. ricini*), *G. orientalis*, *C. longimanus,* and *Euconocephalus* sp. contain high percentages of protein (ranging from 48.64% to 69.38% in dry weight) and are rich in various essential amino acids [12, 48, 51]. They also contain beneficial unsaturated fatty acids [12, 48, 51–54]. *T. papillosa*, *C. longimanus*, *Euconocephalus* sp., *B. mori,* and *S. ricini* contain abundant minerals such as calcium, iron, phosphorus, and zinc [12]. *S. ricini* contains abundant vitamin A and vitamin B2 [12]. However, the local consumption of edible insects was not for nutrition but for taste. The local populace was unaware of the nutritional and health benefits of eating insects. Therefore, improving their awareness of entomophagy’s nutritional benefits could help promote edible insects as a regular source of nutrients in their diet [55, 56].

### Insect therapy

The locals generally used cockroaches and antlions to treat the common cold in infants. In ancient Chinese medicine books, *P. americana* is mentioned as being used to treat malnutrition in children, as well as furuncles, tonsillitis, carbuncles, and snake bites, while antlion was used to treat urolithiasis, malaria, scrofula, and chronic gangrene [12, 57]. As a folk medicine, *P. americana* is used to treat ear pain in Burkina Faso [58] and asthma in Latin America [59]. It is also used to treat asthma, toothaches, and bronchitis by the Amerindians of the Amazon (cited in [57]). In South India, people use crude ash liquor from *P. americana* to cure bladder stones [60]. In Northeast India, antlions are crushed to treat warts [61], while antlion paste is externally applied to cure boils and warts in Nagaland [62]. Antlions are also used for beriberi, whooping cough, and gonorrhea in Japan [16]. Pharmacological studies have found a variety of chemical components in *P. americana* that have antitumor, immunity regulating, liver protecting, tissue repair promoting, anti-inflammatory, and analgesic effects [12]. Antlions contain long-chain fatty acids, fatty acid esters, polypeptides, flavonoids, and alkaloids [63], and mouse experiments have indicated that the components of antlions play a role in enhancing immunity, relieving pain and inflammation [64–66]. Recently, *P. americana* and antlions have been used to produce a variety of medicines that treat gastrointestinal, respiratory, and hepatic diseases in China [12, 67, 68].

Four wasp species and a bamboo bee were used to produce medicinal liquors to treat rheumatism and relieve fatigue. The use of wasps for similar medicinal purposes has been reported in other areas of China. For example, medicinal liquors produced with *V. ducalis*, *V. affinis,* and *Vespa mandarinia* have reportedly been used to treat rheumatism, scapulo-humeral peri-arthritis, sciatica, numbness of the limbs, and traumatic injury in Guizhou province [45] and Dehong Dai-Jingpo Autonomous Prefecture in Yunnan province [69]. However, this is the first time the use of *V. bicolor* and *V. basalis* for a medicinal liquor has been documented. This is also true for *X. dissimilis,* as this species is generally used with herbs to treat aphtha, sore throat, and convulsions in infants [70]. These uses may be due to the presence of the complex compounds of wasp venom, which have beneficial functions, including antimicrobial [71, 72], anticancer [73], and anti-inflammatory effects [74].

Insects are an important resource for new drugs and alternative treatments [16, 18, 19, 22, 23, 58, 62]. They contain useful compounds, and different therapeutic practices could generate different disease treatment effects [16]. There is extensive therapeutic knowledge concerning medicinal insects in various regions and ethnic groups [16, 62]. Although modern medicine is pervasive in healthcare systems, folk medicine is commonly used to treat some diseases. As shown in this study, cockroaches and antlions are extremely useful for treating common cold. Furthermore, most local folk medicines are single substance drugs and thus easy to collect. With the exception of some private formulations owned by families, locals are willing to share their folk medicinal information, and thus, it is "common knowledge." Therefore, it is necessary to use ethnographic methods to record these traditional entomo-therapeutic practices and search for compounds with pharmacological activities [16, 18, 62]. To protect local traditional medicinal knowledge and resources, the sustainability of medicinal resources should be the subject of future investigations, including other local animal medicines.

### Availability of edible and medicinal insect resources

In our study area, most of the insects used in local diets and medicine were collected from the natural environment, except for two artificially bred species, *S. ricini* and *B. mori*. Antlions and cockroaches were readily available as they were common in the surroundings. In contrast, the other insects were only seasonally or occasionally harvested.

According to the respondents, the use of chemical pesticides to protect agricultural products was the primary factor resulting in a reduction in *Euconocephalus* sp. and *G. orientalis* populations in farmlands, while habitat loss was a devastating blow to *A. chamaeleon*. In this regard, decreased availability may constrain the consumption of these edible insects. However, it should be noted that local people seldom consider the sustainable availability of edible insects as they are not a primary food source. It is easier to obtain conventional foods using advanced and modern agricultural planting and animal breeding techniques.

Most edible and medicinal insects are still harvested from the wild [1, 10, 75]. However, the limited availability (e.g., lower quantity and seasonal dependence) of wild insects may not meet the demand for insects as food and medicine [23, 75]. Excessive collection in the wild also threatens species diversity and ecological security [11]. Still, farming insects can produce a large yield in a short time, which is conducive to the harvest, storage, inspection, batch formation, and standardization of products [1, 11]. Insect farming may also offer a new source of sustainable food and medicine [1, 75]. In our study area, *Euconocephalus* sp., *G. orientalis,* and *C. longimanus* were the most preferred and culturally important species. In addition to their phytophagous diet and nonaggressive nature, these insects have the potential to be raised as mini-livestock, providing a stable supply for local consumers. It is also worth noting that *G. orientalis* was used as a live feed for pet birds in Guiyang City in Guizhou province, and the farming of this species may generate considerable income [46].

## Conclusions

This study documented a variety of edible and medicinal insects used by local people. Due to the long-term use of wild insects, local villagers have accumulated considerable local and traditional knowledge. However, this knowledge is positively correlated with age, which may indicate a risk of losing it in the future. Hence, it is imperative to collect, sort, and maintain this valuable knowledge.

Entomophagy has enriched local dietary diversity. However, the respondents in this study emphasized that insects were more commonly consumed in the past. While the palatability of edible insects was the main motivation for their consumption, improving locals’ awareness of their nutritional benefits may promote entomophagy as a regular source of nutrient intake. The decline in entomophagy may also be attributed to the limited availability of edible insects. A more sustainable supply, such as insect farming, may be required to maintain local entomophagy.

Medicinal insects are a part of local folk medicine. In this study, four wasp species and a bamboo bee were used for the treatment of rheumatism, and three cockroach species and an unidentified antlion species were used to treat the common cold. Applying pharmacological and chemical techniques to identify various biologically active substances in these insects should be pursued [12, 71–74], as the combined use of traditional medicinal knowledge and modern science may promote the development of novel drugs and alternative treatments [16, 22].

## Data Availability

All data generated or analyzed during this study are included in this published article.

## Abbreviation

ZAR: Zhuang Autonomous Region
